# Diagnostic Significance of Metagenomic Next‐Generation Sequencing in Immunocompromised Patients With Suspected Pulmonary Infection

**DOI:** 10.1111/imm.13911

**Published:** 2025-02-23

**Authors:** Xi Zheng, Wei Zou, Shumei Zou, Jia Ye, Zhenming Bao, Yingfang Song

**Affiliations:** ^1^ Department of Pulmonary and Critical Care Medicine Tiantai People's Hospital Taizhou China; ^2^ Department of Pulmonary and Critical Care Medicine Dongfang Hospital, School of Medicine, Xiamen University (The 900th Hospital of the Joint Logistic Support Force of the People's Liberation Army of China Fuzong Clinical College of Fujian Medical University) Fuzhou China

**Keywords:** diagnostic performance, immunocompromised patients, metagenomic next‐generation sequencing, organ transplantation, suspected pulmonary infection

## Abstract

Immunocompromised hosts are highly vulnerable to lung infections, but the efficacy of traditional diagnosis is unsatisfactory. Metagenomic next‐generation sequencing (mNGS) has high throughput and broad coverage. Its value in different types of immunocompromised patients has yet to be fully explored. Therefore, the study aims to evaluate the value of mNGS in immunocompromised patients. Clinical data from immunocompromised patients with suspected pulmonary infection (PI) (September 2018–2021) were retrospectively analysed. Patients were categorised into PI (87 cases) and non‐pulmonary infection (NPI, 14 cases) groups. The diagnostic performance between mNGS and conventional microbiological tests (CMTs) was compared. Subgroup analyses were also conducted based on whether the patients received organ transplantation, including the comparison of the diagnostic performance of mNGS and culture and the spectrum of characteristics among them. mNGS demonstrated significantly elevated diagnostic sensitivity (*p* < 0.001) over traditional methods, with a pronounced advantage in identifying mixed PIs (*p* < 0.05). Among immunocompromised cohorts, mNGS outperformed cultures, showing higher positivity rates in both organ transplant (*p* < 0.001) and non‐transplant patients (*p* < 0.001). Mixed infections, predominantly bacterial–fungal, were more prevalent in transplant recipients with reduced lymphocytes and CD4^+^ T cells. Pathogen profiles differed, with *Pneumocystis jirovecii*, *Cytomegalovirus*, and *Pseudomonas aeruginosa* predominating in organ transplant recipients, and *P. jirovecii*, *P. aeruginosa* , *Streptococcus pneumoniae* and *Streptococcus pallidum* in non‐transplant individuals. mNGS is valuable in diagnosing PI and mixed infections in immunocompromised patients, which may be particularly suitable for identifying mixed infections in patients with organ transplants and low lymphocyte and CD4^+^ T lymphocyte counts.

## Graphical abstract

Our study evaluates the diagnostic value of metagenomic next‐generation sequencing (mNGS) in immunocompromised patients with suspected pulmonary infections. mNGS showed superior sensitivity over conventional methods, especially in identifying mixed infections in organ transplant recipients and patients with low lymphocyte counts. Pathogen profiles differed significantly between transplant and non‐transplant patients, highlighting the distinct diagnostic advantage of mNGS in this population.

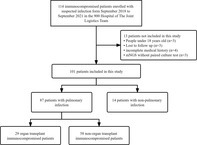

## 1 Introduction

Due to innate and adaptive immune system dysfunction, immunocompromised hosts are more susceptible to various infections caused by pathogenic microorganisms, especially lung infections [1]. In immunocompromised patients, lung infection can cause significant morbidity and mortality [2]. So, it is vital to make a precise and timely diagnosis of pulmonary infection (PI) for proper anti‐infection treatment, good prognosis and reduced mortality [3]. However, the pathogen spectrum of pneumonia in immunocompromised patients is broad, complex and diverse, and mixed pathogens are often present at the same time due to the destruction of the host's immune function [4, 5]. Traditional microbiological methods can be extremely challenging and can not meet the clinical requirements. Only 30%–40% of pathogens can be detected by traditional pathogen tests [6], which is limited by a time‐consuming process and limited pathogen detection and low positivity rate [7].

Metagenomic next‐generation sequencing (mNGS) has been extensively applied in infection with high‐throughput sequencing, broad pathogen coverage, and short turnaround time. However, there is rare research on the application of mNGS in immunocompromised patients with PI [8, 9, 10, 11, 12, 13, 14]. Studies with a large sample size are lacking. Few studies assess the diagnostic value of mNGS for mixed infections in immunocompromised patients. Little is known about the diagnostic performance of mNGS and the spectrum of characteristics in immunocompromised patients with and without organ transplantation. Therefore, we conducted this study to further explore the value of mNGS to provide a decision‐making reference in clinical practice.

## 2 Material and Methods

### 2.1 Study Design and Participants

**FIGURE 1:**
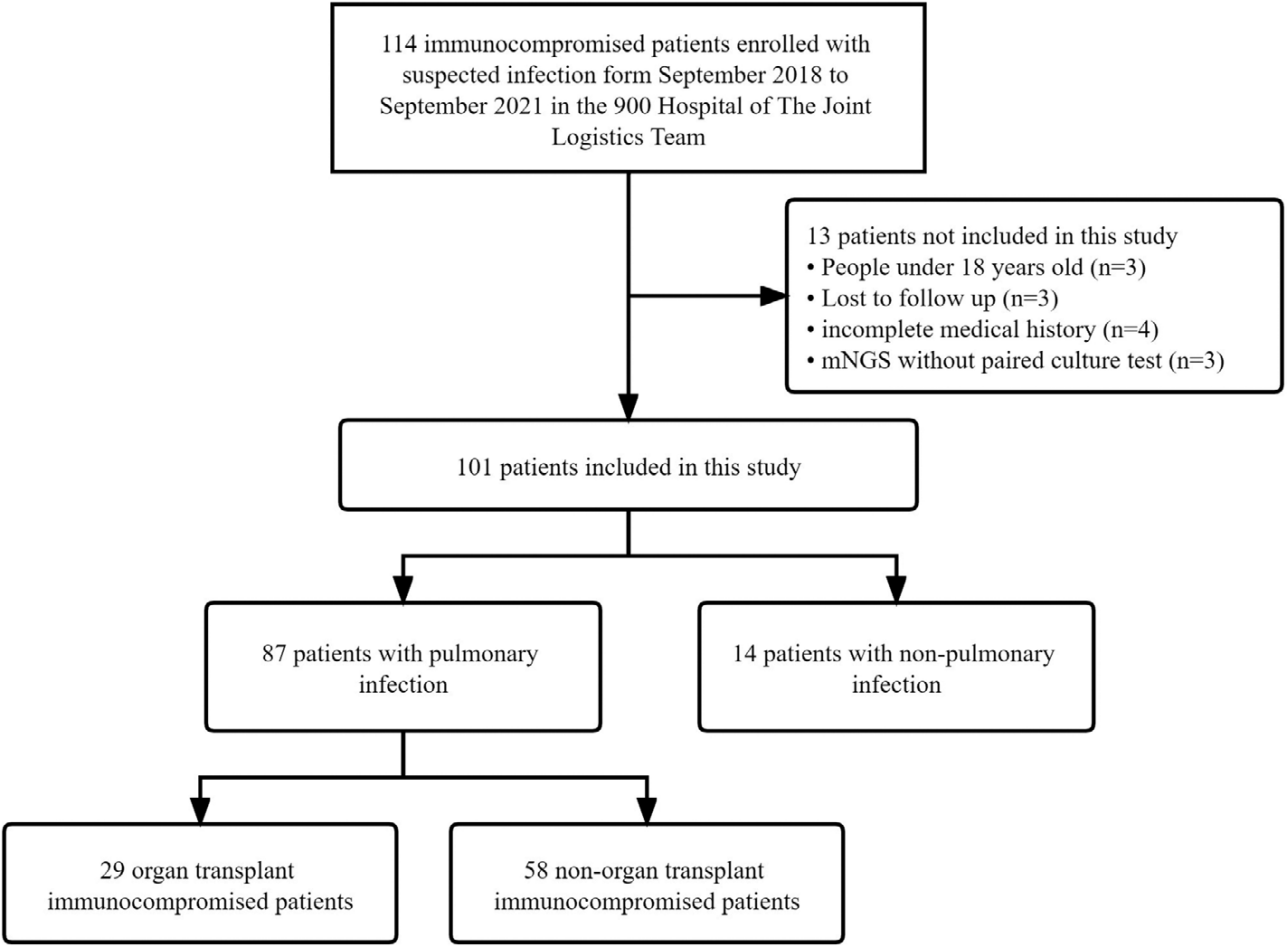
Flow diagram of the study. One hundred fourteen patients were retrospectively analysed and 101 patients were enrolled for further detailed analysis, finally after excluding 13 patients who did not meet the inclusion criteria. There were 87 immunocompromised patients with pulmonary infection and 14 immunocompromised patients with non‐pulmonary infection. Besides, there were 29 organ transplant patients and 58 non‐organ transplant patients among the 87 patients with pulmonary infection.

One hundred fourteen immunocompromised patients with suspected PI admitted to the Department of Pulmonary and Critical Care Medicine at the 900th Hospital of The Joint Logistics Team from September 2018 to September 2021 were retrospectively analysed. In total, 101 patients were enrolled finally for further detailed analysis by inclusion/exclusion criteria in the study and categorised into two groups defined as PI [11] and non‐pulmonary infection (NPI) according to a clinical composite diagnosis (Figure 1). All enrolled patients' bronchoalveolar lavage fluid (BALF) specimens were subjected to microbial culture as well as mNGS in a pairwise manner. The present study was approved by the Ethics Committee of The 900 Hospital of the Joint Service Support Force of the People's Liberation Army of China (No. 2023‐103). Informed consent was obtained from all participants.

The inclusion criteria were as follows: (1) patients aged more than 18 years, (2) immunocompromised patients with suspected PI; and BALF mNGS and other conventional microbiological tests (CMTs) were conducted simultaneously. The definition of immunocompromised was compliant with the latest article published in Chest, 2020 [1]. The diagnosis of PI was defined by the Infectious Diseases Society of America (IDSA)/American Thoracic Society (ATS) criteria [15]. Patients with incomplete clinical and laboratory data were excluded.

### 2.2 The Process of Bronchoscopy and Specimen Collection

BALF was collected by experienced bronchoscopists abiding by the standard procedure. According to chest CT, the bronchus of the lesion site was intubated by a bronchoscope under aseptic conditions and then instilled with sterile normal saline. Finally, the saline was suctioned out under negative pressure. To avoid contamination, we discharged the first 20 mL, then divided the remaining samples into two parts and subjected them to mNGS and culture in a pairwise manner.

Other relevant samples (e.g., blood and sputum) were also collected in this study for CMTs, that is, culture, galactomannan (GM) test (Bio‐Rad Laboratories, Hercules, CA, USA), *Cryptococcus* capsular polysaccharide antigen (CrAg) (IMMY, Norman, OK, USA), and pulmonary histopathology when clinically assessed as necessary. Our comprehensive culturing protocols encompassed multiple specialised media and conditions to maximise pathogen detection across diverse microbial populations. Samples were simultaneously processed using aerobic and anaerobic culture conditions, with inoculation performed within 2 h of sample collection. For bacterial isolation, we utilised blood agar (5% sheep blood), chocolate agar, and MacConkey agar, incubated at 35°C–37°C for 48–72 h. Anaerobic cultures were processed using specialised thioglycollate broth and Brucella blood agar, incubated in an anaerobic chamber (GasPak system) at 35°C–37°C for up to 10 days. Fungal cultures employed Sabouraud dextrose agar, incubated at 30°C and 37°C for 14 days, with weekly subculturing to capture slow‐growing organisms. Each culture batch included positive and negative control samples to ensure method reliability. Microbial identification was performed using a combination of morphological examination, biochemical testing (API systems), and MALDI‐TOF mass spectrometry for precise taxonomic classification, with a minimum of three independent observers cross‐validating identification results.

### 2.3 Pathogenic Analysis and Criteria for a Positive Result

Once the sample was obtained, mNGS was performed depending on the actual condition and wishes of the patients. The sample was sent to Xunminkang Biotechnology Co. Ltd. or Jieyi Biotechnology Co. Ltd. for sequencing, performing nucleic acid extraction, library preparation, bioinformatics analysis, and interpretation and reporting [16]. DNA extraction and library preparation were conducted on the NGS Automatic Library Preparation System (Cat. MAR002, MatriDx Biotech Corp. Hangzhou, China). Reagents included: Nucleic Acid Extraction Kit (Cat. MD013, MatriDx Biotech Corp. Hangzhou, China), Cell‐free DNA Library Preparation Kit (blood samples) (Cat. MD007, MatriDx Biotech Corp. Hangzhou, China) and Total DNA Library Preparation Kit (other samples) (Cat. MD001T, MatriDx Biotech Corp. Hangzhou, China). Libraries were pooled and then sequenced on an Illumina NextSeq500 system using a 75‐cycle sequencing kit. High‐quality sequencing data were generated by removing low‐quality and short (length < 35 bp) reads, followed by computational subtraction of human host sequences mapped to the human reference genome (hg19) using Burrows‐Wheeler alignment. The data remaining after the removal of low‐complexity reads were classified by simultaneous alignment to four microbial genome databases consisting of viruses, bacteria, fungi and parasites. The classification reference databases were downloaded from the National Center Biotechnology Information (ftp://ftp.ncbi.nlm.nih.gov/genomes/). RefSeq contains 4189 whole‐genome sequences of viral taxa, 2328 bacterial genomes or scaffolds, 199 fungi related to human infection and 135 parasites associated with human diseases. A total of 50 bp reads were obtained for each sample. Each trial included internal, negative and positive controls. Internal parameters are specific molecular tags that are placed in the sample before nucleic acid extraction to track the entire process and to control the quality of DNA. The detection results of negative control products should be that no pathogens were detected. If there are relevant pathogens detected, it indicates that there may be DNA pollution sources in the environment. Positive samples contained specific microbic DNA. The CMTs were conducted by our Microbiology Laboratory according to the standard procedure.

Results from mNGS data were considered positive if one of the following criteria were met: Bacteria (mycobacteria excluded), fungi and viruses: its relative abundance was more than 30% at the species level or the coverage rate of bacteria or viruses scored 10‐fold higher than any other microorganism or 5‐fold higher than any other fungus; *Mycobacterium tuberculosis* (MTB): there was at least one read mapped to the species or genus level; non‐tuberculosis mycobacteria (NTM) ranks among the top 10 in the list of bacteria; it was considered positive that the mNGS reads number was more than 50 from a single species when mNGS and culture identified the same microbe [13, 17]. Recognising the critical challenge of non‐uniform guidelines in mNGS interpretation, our research team developed a rigorous, multi‐step standardisation protocol to mitigate potential interpretative bias. We implemented a comprehensive framework that includes predefined criteria for pathogen significance, involving a three‐tier expert review process with independent microbiologists, infectious disease specialists, and bioinformatics experts. Our standardisation strategy incorporated quantitative thresholds for microbial detection, considering factors such as relative abundance, read depth, taxonomic specificity, clinical context, patient‐specific risk factors, underlying conditions and potential contamination sources, ensuring consistent interpretation across different clinical scenarios. Our protocol includes mandatory cross‐validation by at least two independent reviewers, with discrepancies resolved through comprehensive case discussion and a consensus approach.

### 2.4 Clinical Evaluation

We regarded the clinical composite diagnosis of the patient as the reference standard, which was made by at least two experienced respiratory physicians based on medical records, clinical manifestations, imaging results, the results of CMTs and mNGS, and outcomes after anti‐infective treatments. Mixed infection was defined as two or more pathogens' infections and non‐mixed infection was described as a single or unknown pathogen infection. According to the clinical composite diagnosis, patients were divided into the PI and NPI groups.

### 2.5 Statistical Analysis

SPSS 26.0 software was used for statistical analysis. Continuous numerical variables in accordance with the normal distribution were presented as the means ± SDs, median (upper and lower quartiles) in accordance with non‐normal distribution. Categorical variables were recorded as ratios or percentages. Normal and non‐normal distributions use *t*‐tests and nonparametric tests to determine, respectively. The McNemar test, Fisher exact test or Pearson chi‐square test was performed for discrete variables when appropriate. Categorical variables were reported as frequencies and percentages and analysed using the Fisher exact test or Pearson chi‐square test according to sample sizes. The Fisher exact test proves most valuable for small sample sizes (*n* < 30) and the Pearson chi‐square test, conversely, is most appropriate for larger sample sizes (*n* ≥ 30). The McNemar test was used to compare the positive rate of culture and mNGS. *p* < 0.05 was defined as a significant difference.

## 3 Results

### 3.1 Sample and Patient Characteristics

Among the 101 patients, there were 87 patients with PI and 14 patients with NPI. Patient characteristics and baseline are shown in Table 1. In the PI group, 74.7% (65 out of 87) of patients were male, and the mean age was 57. In the NPI group, 50.0% (7 out of 14) of patients were male, and the mean age was 51.6. The level of the inflammation indicator C‐reactive protein (CRP) and procalcitonin was significantly higher in the PI group than in the NPI group (*p* < 0.05), but the counts of white blood cells (WBCs) were comparable for patients with PI and those with NPI, which could not be used reliably to distinguish PI from NPI. Lymphocyte counts were lower in patients with PI compared to patients with NPI (*p* < 0.05).

**TABLE 1:** Patient characteristics and baseline of the two groups.

	PI group (*n* = 87)	NPI group (*n* = 14)	*p*
Characteristics			
Age, years	57.0 ± 15.1	51.6 ± 13.9	0.893
Male, *n* (%)	65 (74.7%)	7 (50.0%)	0.114
Laboratory parameters			
WBC (10^9^/L)	8.2 (5.0, 10.9)	7.6 (4.7, 9.0)	0.318
Lymphocyte (10^9^/L)	0.8 (0.4, 1.3)	1.6 (0.8, 1.9)	0.032*
Hb (g/L)	104.7 ± 23.9	110.9 ± 20.6	0.760
PLT (10^9^/L)	220.0 ± 109.1	212.1 ± 103.7	0.847
Alb (g/L)	32.2 ± 7.1	37.7 ± 6.3	0.984
APTT (s)	30.1 (26.6, 33.3)	28.3 (29.8, 30.8)	0.910
CRP (mg/L)	72.0 (41.4, 135)	10.4 (7.2, 36.7)	0.001*
PCT (μg/L)	0.9 (0.2, 3.6)	0.05 (0.05, 0.13)	0.001*

Abbreviations: Alb, albumin; APTT, activated partial thromboplastin time; CRP, C‐reactive protein; Hb, haemoglobin; PLT, platelet count; Scr, serum creatinine; PCT, procalcitonin; WBC, white blood cell.

* *p* < 0.05 was considered statistically significant.

### 3.2 Diagnostic Performance Comparison of mNGS and Culture

#### 3.2.1 Comparison of Diagnostic Performance of BALF mNGS and Culture for Differentiating PI From NPI

**FIGURE 2:**
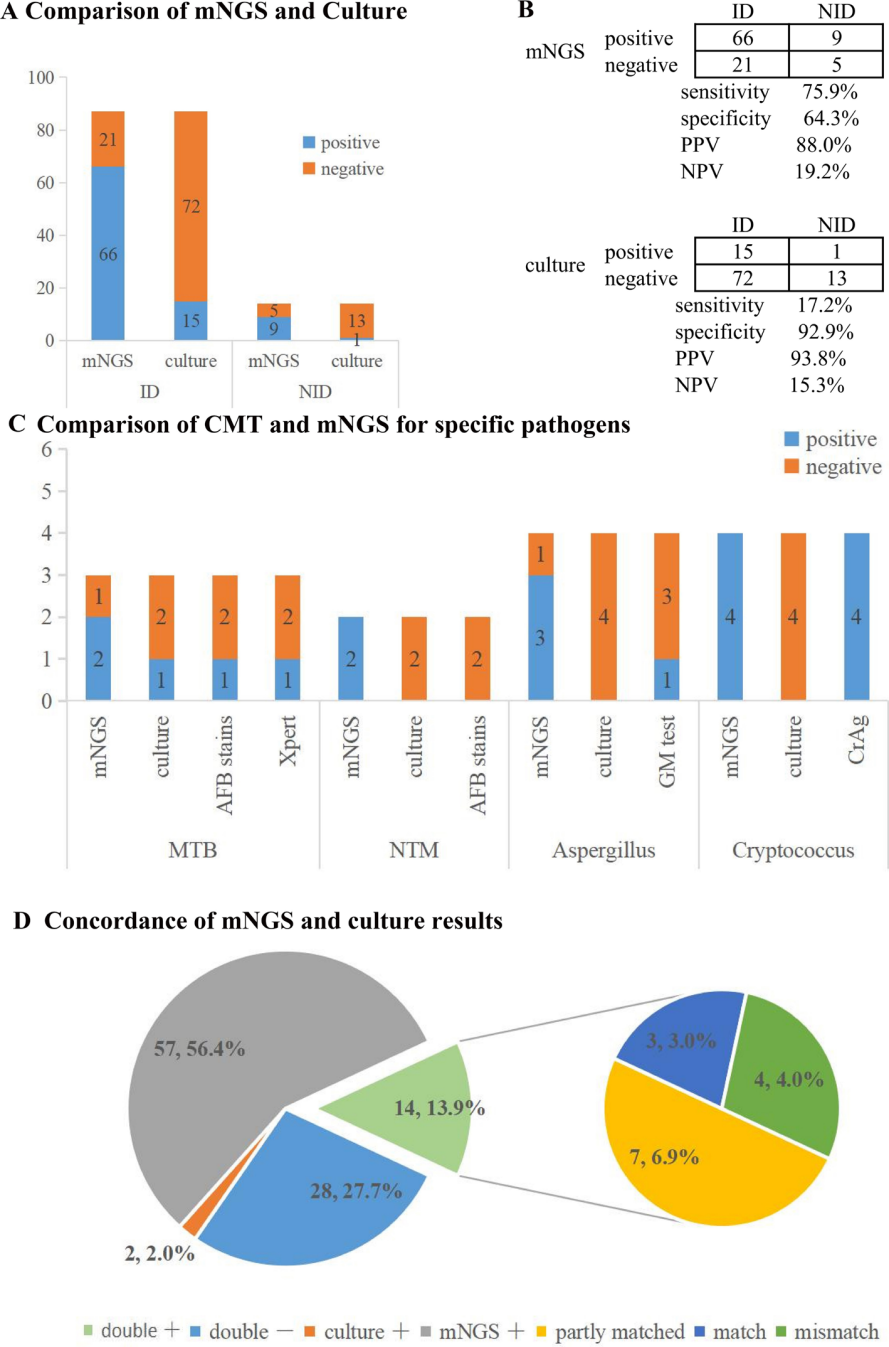
Positivity rate comparison and diagnostic performance and concordance analysis. (A) Positivity rate comparison between mNGS and culture for PI (*n* = 87), NPI (*n* = 14). The number of positive samples (*y*‐axis) for pairwise mNGS and culture in the two groups (*x*‐axis). (B) Contingency tables formatted in a 2 × 2 manner reveal the performance of mNGS and culture in the diagnosis of PI. mNGS increased the sensitivity rate by approximately 58% in comparison with that of culture (75.9% vs. 17.2%; *p* < 0.001), while the specificity difference was not significant. (C) Comparison of CMTs and mNGS for specific pathogens. The number of positive samples (*y*‐axis) for pairwise mNGS and CMTs is plotted against the MTB, NTM, *Aspergillus* and *Cryptococcus* infection groups (*x*‐axis). (D) Pie chart demonstrating concordance between BALF mNGS and culture for pathogen detection. AFB, acid‐fast bacteria; CrAg, *Cryptococcus* capsular polysaccharide antigen; GM, galactomannan; mNGS, metagenomic next‐generation sequencing; MTB, *Mycobacterium tuberculosis* ; NPI, non‐pulmonary infection; NTM, non‐tuberculous mycobacteria; NPV, negative predictive value; PI, pulmonary infection; PPV, positive predictive value; Xpert, xpert *Mycobacterium tuberculosis* /rifampicin resistance.

The positivity rates of mNGS and culture for the PI and NPI groups are demonstrated in Figure 2A. One hundred one patients were enrolled to analysing the diagnostic efficiency for differentiating PI from NPI. As expected, the sensitivity rate of mNGS was significantly higher than that of the culture method (75.9%, 95% CI, 65.3%–84.1% vs. 17.2%, 95% CI, 10.3%–27.2%; *p* < 0.001), while there is no statistical significance in specificity difference (64.3%,95% CI, 35.6%–86.0% vs. 92.9%,95% CI, 64.2%–99.6%; *p* = 0.219) (Figure 2B). For the false‐positive cases detected by mNGS in the NPI group, possible reasons included potential contaminants, colonisation, and other factors such as Inflammatory processes (Table 2). The negative predictive value (NPV) and positive predictive value (PPV) of diagnosing infectious disease by mNGS were 19.2% and 88.0%, respectively, with the negative and positive likelihood ratios being 0.37 and 2.13. The NPV and PPV achieved using BALF culture were 15.3% and 93.8%, respectively, with the negative and positive likelihood ratios being 0.89 and 2.42. Besides, for specific pathogens identified, the sensitivities for detecting MTB, NTM, *Aspergillus* and *Cryptococcus* by mNGS were 66.7%, 100%, 75.0% and 100%, respectively. For MTB, NTM and *Aspergillus* infection, mNGS was superior to CMTs, and CrAg is comparable to mNGS in detecting *Cryptococcus*, although the difference was not statistically significant, possibly owing to the small sample size (Figure 2C).

**TABLE 2:** Analysis of the false‐positive cases detected by mNGS in the NPI group.

Pathogens detected only by mNGS in the NPI group				
Sample no.	Patient no.	Diagnosis	mNGS result	Possible explanation
BALF‐1	PT‐1	Lung cancer	*Rothia mucilaginosa*	Likely contamination
BALF‐3	PT‐3	Lymphoma	*Pseudomonas aeruginosa*	Likely colonisation
BALF‐4	PT‐4	Lymphoma	Epstein–Barr virus	Possible cause of lymphoma
BALF‐6	PT‐6	Fever of unknown origin; liver transplantation	Torque teno virus	Potential cause of inflammation
BALF‐9	PT‐9	Cryptogenic organising pneumonia; post‐renal transplantation	Torque teno virus	Potential cause of inflammation
BALF‐10	PT‐10	Cryptogenic organising pneumonia; post‐renal transplantation	Torque teno virus	Potential cause of inflammation
BALF‐11	PT‐11	Dermatomyositis	*A. baumannii*	Likely colonisation
BALF‐12	PT‐12	During radiotherapy for uterine malignant tumours	*Ralstonia mucilaginosa*	Likely colonisation
BALF‐13	PT‐13	ABPA	*Pseudomonas aeruginosa*	Likely colonisation

Abbreviations: BALF, bronchoalveolar lavage fluid; PT, patient.

#### 3.2.2 Concordance Between mNGS and Culture for Pathogen Detection

In this study, mNGS and culture were positive in 14 (14 out of 101, 13.9%) cases and negative in 28 (28 out of 101, 27.7%) cases. A total of 57 cases were positive by mNGS only (56.4%), while there were only 2 cases (2 out of 101, 2.0%) positive by culture only. For the 14 double‐positive cases, 3 cases were found to be wholly matched and 4 cases were totally mismatched. A total of seven cases were found to be ‘partly matched’ meaning that at least one detected pathogen was overlapped between mNGS and culture (Figure 2D).

### 3.3 Diagnostic Performance of mNGS and CMTs in Immunocompromised Patients With Mixed PI

In 87 immunocompromised patients with PI, the comparison of mNGS and CMTs in the diagnosis of mixed PI is presented in Table 3. Regarding the clinical composite diagnosis as a reference, mNGS had a much higher sensitivity in the diagnosis of mixed PI than that of CMTs (61.7%, 95% CI, 46.4%–75.1% vs. 12.8%, 95% CI, 5.3%–26.4%; *p* < 0.05) and the difference in specificity was not statistically significant.

**TABLE 3:** Diagnostic performance of mNGS and CMTs in the diagnosis of mixed pulmonary infection.

	Sensitivity	Specificity
	% (95% CI)	% (95% CI)
mNGS	61.7 (46.4, 75.1)^a^	95.0 (81.8, 99.1)
CMTs	12.8 (5.3, 26.4)	97.5 (85.3, 99.9)

^a^ mNGS vs. CMTs, *p* < 0.05.

### 3.4 Comparison of Clinical Characteristics Between Mixed and Non‐Mixed PI

As illustrated in Table 4, there were 47 patients with mixed infection and 40 patients with non‐mixed infection among the 87 patients with PI. There were significant differences between mixed infection and non‐mixed infection in terms of WBC (*p* = 0.034), lymphocyte (*p* = 0.024), CD4^+^ T lymphocyte counts (*p* = 0.002) and the number of patients with organ transplantation (*p* = 0.048), which could be considered to be associated with mixed infection. We found that organ transplant patients were more susceptible to mixed infection and that lymphocyte and CD4^+^ T lymphocyte counts were lower in patients with mixed infection compared to patients with non‐mixed infection. Although WBC counts were lower than those of patients with mixed infection, they were within the normal range.

**TABLE 4:** Comparison of clinical characteristics between mixed and single pulmonary infections.

	Mixed infection	Non‐mixed infection	*p*
	*n* = 47	*n* = 40	
Characteristics			
Age, years	53.8 ± 16.6	60.7 ± 12.4	0.31
Gender			0.955
Male, *n* (%)	35 (74.5%)	30 (75.0%)	
Female, *n* (%)	12 (25.5%)	10 (25.0%)	
Laboratory parameters			
WBC (10^9^/L)	7.1 (4.5, 10)	8.9 (7.1, 11.7)	0.034*
Lymphocyte (10^9^/L)	0.6 (0.3, 1.0)	1.0 (0.6, 1.6)	0.024*
Hb (g/L)	101.0 ± 24.6	109.1 ± 22.6	0.117
PLT (10^9^/L)	201.9 ± 101.3	241.3 ± 115.3	0.093
Alb (g/L)	31.6 (27.3, 36.1)	31.4 (29.9, 37.4)	0.327
APTT (s)	29.9 ± 5.7	31.7 ± 3.8	0.086
CRP (mg/L)	68.3 (48.8, 130)	74.8 (40.8, 135.0)	0.692
PCT (μg/L)	0.9 (0.18, 2.4)	0.8 (0.2, 7.5)	0.361
CD4 (*n*/μL)	195 (73, 410)	449 (245, 599)	0.002*
CD8 (*n*/μL)	198 (96.5, 401)	229 (150, 423)	0.314
Type of immunocompromised status, *n* (%)			0.048
Organ transplantation	20 (42.6%)	9 (22.5%)	
Non‐organ transplantation	27 (57.4%)	31 (77.5%)	

Abbreviations: Alb, albumin; APTT, activated partial thromboplastin time; CRP, C‐reactive protein; Hb, haemoglobin; PCT, procalcitonin; PLT, platelet count; Scr, serum creatinine; WBC, white blood cell.

* *p* < 0.05 was considered statistically significant.

### 3.5 Comparison of Diagnostic Performance of BALF mNGS and Culture in Organ Transplant Immunocompromised Patients

**FIGURE 3:**
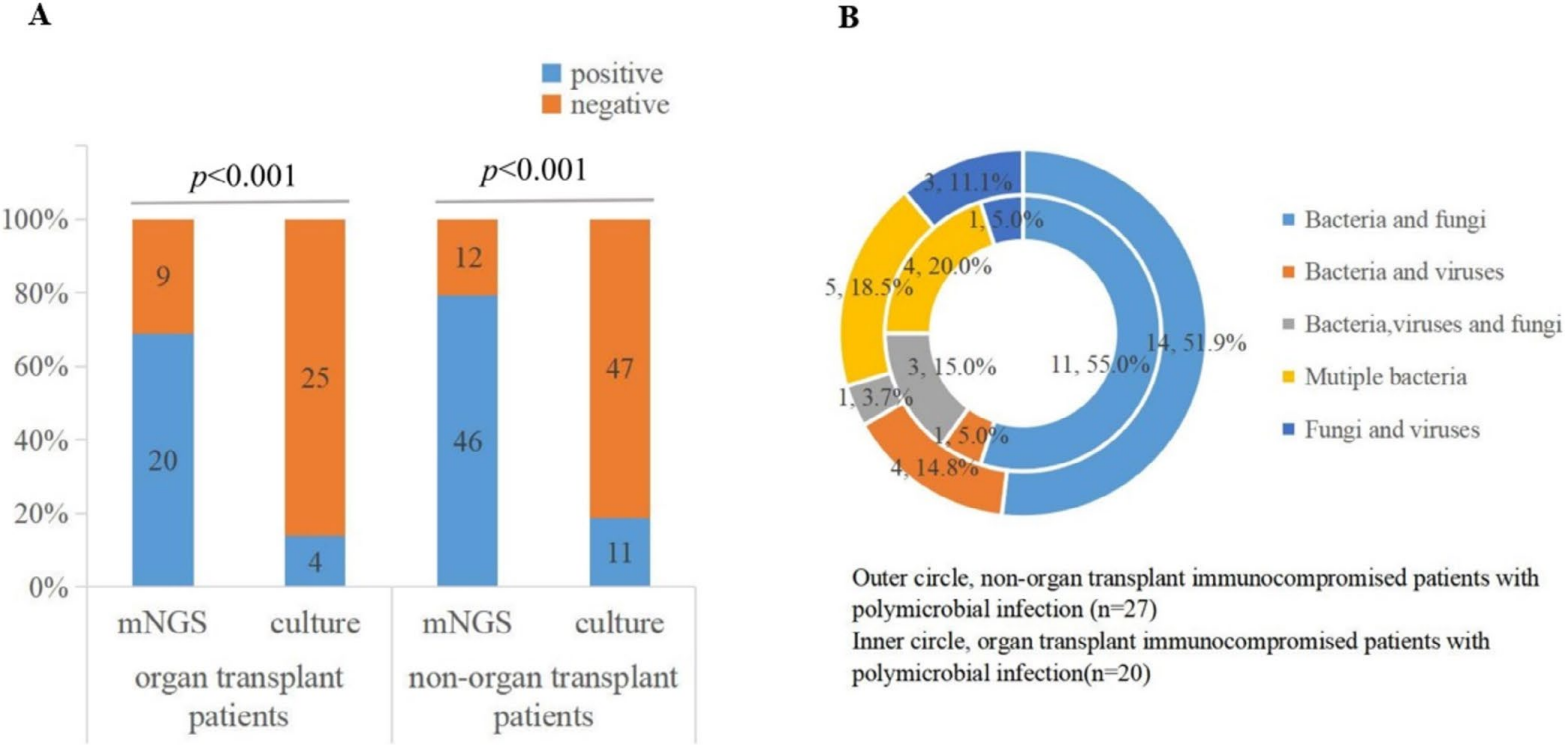
Diagnostic performance of mNGS and distribution of polymicrobial infection in patients with and without organ transplantation. (A) The comparisons of positive rates (*y*‐axis) for pairwise mNGS and culture between immunocompromised patients with and without organ transplantation (*x*‐axis) (*p* < 0.001). (B) Distribution of different types of polymicrobial infection between immunocompromised patients with and without organ transplantation.

There were 29 organ transplant patients and 58 non‐organ transplant patients among the 87 patients with PI. As was shown in Figure 3A, the positivity rate of mNGS was higher than that of culture between organ transplant immunocompromised patients and non‐organ transplant immunocompromised patients (69.0% [20/29], 95% CI, 49.1%–84.0% vs. 13.8 [4/29], 95% CI, 4.5%–32.6%; 79.3% [46/58], 95% CI, 66.3%–88.4% vs. 19.0% [11/58], 95% CI, 10.3%–31.8%; *p* < 0.001). The conclusion we draw above is that organ transplant patients were more susceptible to mixed infection. Therefore, further analysis of the proportion of polymicrobial infection in organ transplant patients is shown in Figure 3B. Although no significant difference in types of polymicrobial infection was observed in the immunocompromised patients with or without organ transplantation, the proportion of bacterial–fungal, bacterial–bacterial and bacterial–fungal–viral infections in organ transplant immunocompromised patients was higher than that in non‐organ transplant immunocompromised patients. The most frequent pattern of mixed infection was bacterial–fungal infection, whether in organ transplant immunocompromised or non‐organ transplant immunocompromised patients.

### 3.6 Characteristics of Pathogens in Organ Transplant Immunocompromised Patients

**FIGURE 4:**
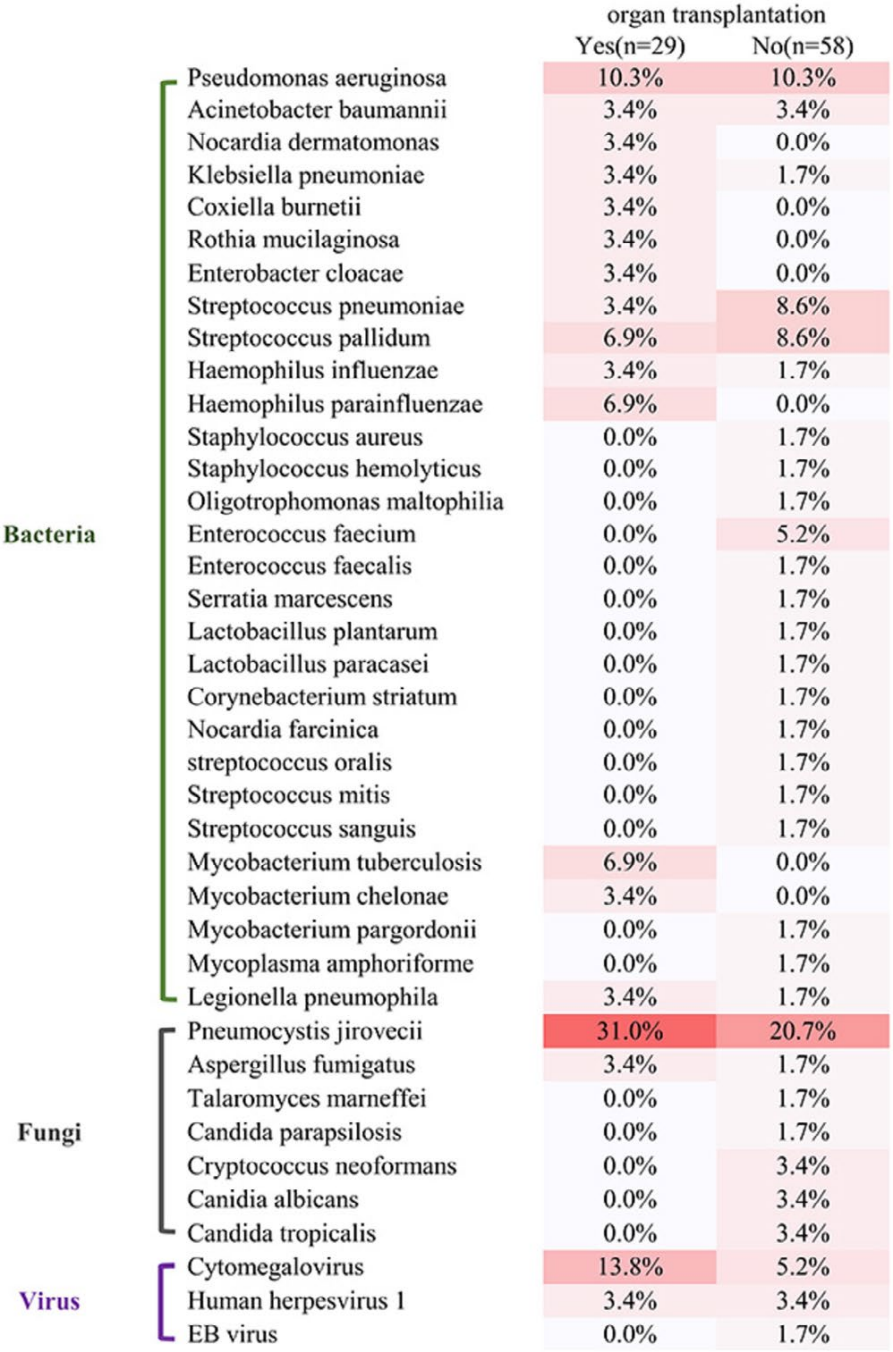
The pathogens' spectrum by mNGS based on the clinical composite diagnosis between immunocompromised patients with and without organ transplantation.

The most common pathogens in organ transplant immunocompromised patients included *Pneumocystis jirovecii* (9), *Cytomegalovirus* (4) and *Pseudomonas aeruginosa* [18], while in non‐organ transplant immunocompromised patients, *P. jirovecii* (12), *P. aeruginosa* (6), *Streptococcus pneumoniae* (5) and *Streptococcus pallidum* (5) were the leading pathogens (Figure 4).

## 4 Discussion

The number of immunocompromised hosts has increased with the development of organ transplantation and the extensive use of antibiotics, hormones and immunosuppressive agents recently. However, the pathogen is diverse and microbial loads are low in immunocompromised patients when infected with pneumonia, so the etiological diagnosis using traditional microbiological methods can be extremely challenging [19, 20]. mNGS has become a complementary tool for clinical diagnosis in recent years [21]. For further evaluating the clinical value of mNGS, we retrospectively conducted a comprehensive study of immunocompromised patients when applying mNGS and further conducted subgroup analyses based on whether the patients received organ transplantation, which had not yet been explored before our study.

In this study, we systematically analysed the diagnostic efficiency for differentiating PI from NPI by mNGS and culture in a pairwise manner and found that mNGS improved the sensitivity rate by approximately 58% compared with culture (75.9% vs. 17.2%; *p* < 0.001), which was significantly higher than in another previous study (15%) [22]. One possible reason is our inclusion of all immunocompromised patients with pathogen diversity and low microbial loads, which caused the sensitivity rate of culture to be relatively low. However, the specificity difference was not significant between mNGS and culture (64.3% vs. 92.9%; *p* = 0.219). The possible reasons for the lower specificity of mNGS were as follows: (1) culture could only detect limited pathogens because of being affected by many factors such as environment and medium, leading to a higher true negative in NPI; (2) mNGS detected pathogens in the samples whose results of the culture were negative, such as the false‐positive rate of mNGS. mNGS can exhibit a high false‐positive rate due to several factors, including contamination from environmental sources, the detection of colonising organisms rather than true pathogens, low‐abundance pathogens being misinterpreted, inflammatory processes releasing microbial DNA and cross‐reactivity with closely related species. These false positives can lead to unnecessary treatments, increased healthcare costs, patient anxiety and misguided clinical decision‐making. Therefore, it is crucial for clinicians to interpret mNGS results with caution, considering these potential pitfalls and correlating findings with the clinical context and additional testing when needed; (3) mNGS could detect all kinds of pathogens at the same time due to its high‐throughput sequencing and broad pathogen coverage. Overall, mNGS was superior to culture in the etiological diagnosis of PI in immunocompromised patients.

Compared with immunocompetent patients, mixed infections were more common in immunocompromised patients [13]. The possibility of polymicrobial infection may be due to the following reasons: (1) the destruction of the host's immune function in immunocompromised patients; (2) the respiratory tract as an open passageway being exposed to a variety of pathogens [4, 5, 23]. CMTs hardly identified mixed infection because of its limitations such as the low positivity rate and limited pathogen detection. On the other hand, mNGS, as a high‐throughput sequencing method, directly detects the nucleic acids of pathogens in samples and can identify a comprehensive pathogen‐detection spectrum in a single assay, including viruses, bacteria, fungi and parasites [24]. That is to say that mNGS could identify multiple pathogens directly in one sample without a prior knowledge of the possible pathogens and additional testing. A growing number of studies have demonstrated that mNGS is better at identifying mixed infection than CMTs such as culture, polymerase chain reaction (PCR) and serological antibody in prior studies [11, 14, 25, 26, 27, 28]. As also demonstrated in our study, mNGS showed a much higher sensitivity in the diagnosis of mixed PI than that of CMTs (61.7% vs. 12.8%, *p* < 0.05). Therefore, mNGS is deemed to benefit immunocompromised patients with a high suspicion of mixed infection. Interestingly, our study showed that immunocompromised patients with organ transplantation were more susceptible to mixed infection. Moreover, immunocompromised patients with mixed infection had significantly lower lymphocyte and CD4^+^ T lymphocyte counts compared to patients without mixed infection. In other words, immunocompromised patients with organ transplantation and advanced immunosuppression are more inclined to be infected by mixed pathogens, which can be explained by the low immunity of these patients. mNGS may be more appropriate for patients with such conditions. So, mNGS could be an especially preferred method for immunocompromised patients with organ transplantation and advanced immunosuppression who are more susceptible to mixed infection.

The immune function of organ transplant patients is obviously impaired due to long‐term immunosuppressive therapy, increasing the chance of infection. Our study is the first to divide immunosuppressed patients based on etiological composition into organ transplant patients and non‐organ transplant patients and analyse the spectrum of characteristics among them. As compared with culture, mNGS had a higher positive rate whether in organ transplant immunocompromised patients or in non‐organ transplant immunocompromised patients (69.0% [20/29] vs. 13.8 [4/29]; 79.3% [46/58] vs. 19.0% [11/58], *p* < 0.001). mNGS has more advantages in detecting pathogens than culture, regardless of which population it detects. Our results revealed that the most frequent pattern of mixed infection was bacterial–fungal infection, whether in organ transplant immunocompromised patients or in non‐organ transplant immunocompromised patients, which is inconsistent with some studies [5, 21, 28, 29]. The possible explanation may be the different populations and samples in different studies. We found that immunocompromised patients with organ transplantation were more susceptible to mixed infection. Patients with mixed PI may develop more serious diseases and are difficult to diagnose. So, a precise and timely diagnosis of mixed PI is a top priority. Our study has proved that mNGS has an absolute advantage in diagnosing mixed PI. mNGS may be more suitable for use in immunocompromised patients with organ transplantation. The pathogen spectra of immunocompromised patients differed between organ transplant patients and non‐organ transplant patients. The most common pathogens in organ transplant immunocompromised patients included *P. jirovecii* (9), *Cytomegalovirus* (4) and *P. aeruginosa* [18]. *P. jirovecii*, as the most common opportunistic pathogens in immunocompromised patients, had the largest proportion (31.0%) in immunocompromised patients with organ transplantation in our study, indicating its high pathogenicity. *P. jirovecii* pneumonia (PCP) poses a potentially direct threat to human health primarily seen in immunocompromised individuals, including organ transplant recipients and PCP occurs in 5%–15% of organ transplant recipients [30]. Therefore, it is very important to diagnose PCP as early as possible. It is noteworthy that our study detected *P. jirovecii* only by mNGS because it could not be obtained in pure culture. In clinical work, Gomori methenamine silver staining is the diagnostic standard for PJP, but the diagnostic sensitivity is rather low [31]. What is worse, the GMS is not conducted in most hospitals. Nevertheless, mNGS had a sensitivity of 100% in diagnosing the infection of *P. jirovecii*, indicating the immense diagnostic value for *P. jirovecii* [32, 33]. Similarly, Lin et al. evaluated the diagnostic value of mNGS of BALF in immunocompromised patients with suspected pneumonia. The results indicated that mNGS had a higher overall detection rate for pathogens compared to CMTs. However, for bacterial and viral infections, the diagnostic accuracy of mNGS was comparable to that of CMTs. Notably, mNGS exhibited higher diagnostic accuracy for fungal detection, particularly in cases of PJP, where mNGS sensitivity was 100% compared to 28% for CMTs. This suggests that while mNGS offers advantages in certain infections, its superiority over CMTs may not be universal across all pathogen types [9]. Therefore, the application of mNGS can contribute to the identification of PJP.

Admittedly, there are some limitations in our research. While mNGS offers comprehensive pathogen detection, its high cost and longer turnaround time compared to some rapid diagnostic tests may limit its routine clinical use. Zhou et al. [34] also noted that despite higher detection rates, the impact of mNGS on clinical outcomes, such as mortality and length of hospital stay, was not significantly different from traditional methods. Thus, the clinical benefits of mNGS need further evaluation to justify its widespread adoption. Also, the retrospective nature of this study is a limitation, as it may introduce inherent biases, such as selection bias and information bias, which could affect the validity of our findings, particularly in a diagnostic context. Additionally, the sample size for subgroup analyses (e.g., organ transplant immunocompromised patients and non‐organ transplant immunocompromised patients) is relatively small. This limits the generalisability of the results to broader populations of immunocompromised patients. Therefore, further prospective studies with a large sample size are needed to explore the clinical value of mNGS in different types of immunocompromised patients on account of their etiological composition. While the study compares mNGS with CMTs, it does not consider other emerging diagnostic techniques, such as PCR‐based methods or serological tests. Further prospective studies are needed to provide a more comprehensive view of where mNGS stands relative to alternative diagnostics. Furthermore, RNA sequencing was not conducted with DNA sequencing at the same time, resulting in the missing of useful supplementary information such as microbial transcriptome changes and RNA viruses. In future works, we aim to further implement parallel DNA and RNA sequencing protocols, focusing on immunocompromised patients with suspected PI to capture nuanced microbial dynamics and transcriptional alterations. By integrating DNA and RNA sequencing, we can potentially identify not just microbial presence but also active infection states, virulence factor expression, and host–pathogen interaction mechanisms, thereby significantly advancing our understanding of complex infectious processes. Moreover, the clinical composite diagnosis of the patient was made by at least two experienced respiratory physicians, so subjective bias is unavoidable. Finally, there is currently a lack of uniform guidance in interpreting mNGS results, which can have an impact on the assessment of mNGS performance to some extent.

In conclusion, our study probed into the diagnostic performance of mNGS in immunocompromised patients. mNGS is a promising diagnostic method for diagnosing PI and mixed infections in immunocompromised patients, especially for immunocompromised patients with organ transplantation and advanced immunosuppression who are more prone to being infected by mixed infections.

## Author Contributions

All authors contributed to the study conception and design. Material preparation, data collection and analysis were performed by X.Z., W.Z., S.M.Z., J.Y., Z.M.B. and Y.F.S. The first draft of the manuscript was written by X.Z., and Y.F.S. commented on previous versions of the manuscript. All authors read and approved the final manuscript.

## Ethics Statement

The present study was approved by the Ethics Committee of The 900 Hospital of the Joint Service Support Force of the People's Liberation Army of China (No. 2023‐103).

## Consent

The authors have nothing to report.

## Conflicts of Interest

The authors declare no conflicts of interest.

## Data Availability

The data sets generated during and/or analysed during the current study are available from the corresponding author on reasonable request.

## References

[1] J. A. Ramirez , D. M. Musher , S. E. Evans , et al., “Treatment of Community‐Acquired Pneumonia in Immunocompromised Adults: A Consensus Statement Regarding Initial Strategies,” Chest 158 (2020): 1896–1911, 10.1016/j.chest.2020.05.598. 32561442 PMC7297164

[2] N. Benito , A. Moreno , J. M. Miro , and A. Torres , “Pulmonary Infections in HIV‐Infected Patients: An Update in the 21st Century,” European Respiratory Journal 39 (2012): 730–745, 10.1183/09031936.00200210. 21885385

[3] J. Kawada , Y. Okuno , Y. Torii , et al., “Identification of Viruses in Cases of Pediatric Acute Encephalitis and Encephalopathy Using Next‐Generation Sequencing,” Scientific Reports 6 (2016): 33452, 10.1038/srep33452. 27625312 PMC5022051

[4] H. L. Fulkerson , M. T. Nogalski , D. Collins‐McMillen , and A. D. Yurochko , “Overview of Human Cytomegalovirus Pathogenesis,” in Methods in Molecular Biology, vol. 2244, ed. A. D. Yurochko (Humana, 2021), 1–18, 10.1007/978-1-0716-1111-1_1. 33555579

[5] J. Legoff , N. Zucman , V. Lemiale , et al., “Clinical Significance of Upper Airway Virus Detection in Critically Ill Hematology Patients,” American Journal of Respiratory and Critical Care Medicine 199 (2019): 518–528, 10.1164/rccm.201804-0681OC. 30230909

[6] D. M. Musher , I. L. Roig , G. Cazares , C. E. Stager , N. Logan , and H. Safar , “Can an Etiologic Agent Be Identified in Adults Who Are Hospitalized for Community‐Acquired Pneumonia: Results of a One‐Year Study,” Journal of Infection 67 (2013): 11–18, 10.1016/j.jinf.2013.03.003. 23523447 PMC7132393

[7] S. Jain , W. H. Self , R. G. Wunderink , et al., “Community‐Acquired Pneumonia Requiring Hospitalization Among U.S. Adults,” New England Journal of Medicine 373 (2015): 415–427, 10.1056/NEJMoa1500245. 26172429 PMC4728150

[8] J. F. Camargo , A. A. Ahmed , M. S. Lindner , et al., “Next‐Generation Sequencing of Microbial Cell‐Free DNA for Rapid Noninvasive Diagnosis of Infectious Diseases in Immunocompromised Hosts,” F1000Research 8 (2019): 1194, 10.12688/f1000research.19766.4. 31814964 PMC6883395

[9] P. Lin , Y. Chen , S. Su , et al., “Diagnostic Value of Metagenomic Next‐Generation Sequencing of Bronchoalveolar Lavage Fluid for the Diagnosis of Suspected Pneumonia in Immunocompromised Patients,” BMC Infectious Diseases 22 (2022): 416, 10.1186/s12879-022-07381-8. 35488253 PMC9052728

[10] T. Pan , R. Tan , H. Qu , et al., “Next‐Generation Sequencing of the BALF in the Diagnosis of Community‐Acquired Pneumonia in Immunocompromised Patients,” Journal of Infection 79 (2019): 61–74, 10.1016/j.jinf.2018.11.005. PMC7133759 30476493

[11] P. Parize , E. Muth , C. Richaud , et al., “Untargeted Next‐Generation Sequencing‐Based First‐Line Diagnosis of Infection in Immunocompromised Adults: A Multicentre, Blinded, Prospective Study,” Clinical Microbiology and Infection 23 (2017): 574.e1–574.e6, 10.1016/j.cmi.2017.02.006. 28192237

[12] J. M. Peng , B. Du , H. Y. Qin , Q. Wang , and Y. Shi , “Metagenomic Next‐Generation Sequencing for the Diagnosis of Suspected Pneumonia in Immunocompromised Patients,” Journal of Infection 82 (2021): 22–27, 10.1016/j.jinf.2021.01.029. 33609588

[13] T. Sun , X. Wu , Y. Cai , et al., “Metagenomic Next‐Generation Sequencing for Pathogenic Diagnosis and Antibiotic Management of Severe Community‐Acquired Pneumonia in Immunocompromised Adults,” Frontiers in Cellular and Infection Microbiology 11 (2021): 661589, 10.3389/fcimb.2021.661589. 34141628 PMC8204719

[14] J. Li , C. E. Zhou , S. C. Wei , et al., “Diagnostic Value of Metagenomic Next‐Generation Sequencing for Pneumonia in Immunocompromised Patients,” Canadian Journal of Infectious Diseases & Medical Microbiology = Journal Canadien Des Maladies Infectieuses et de la Microbiologie Medicale 2022 (2022): 5884568, 10.1155/2022/5884568. PMC9731749 36507192

[15] J. P. Metlay , G. W. Waterer , A. C. Long , et al., “Diagnosis and Treatment of Adults With Community‐Acquired Pneumonia. An Official Clinical Practice Guideline of the American Thoracic Society and Infectious Diseases Society of America,” American Journal of Respiratory and Critical Care Medicine 200 (2019): e45–e67, 10.1164/rccm.201908-1581ST. 31573350 PMC6812437

[16] W. Gu , S. Miller , and C. Y. Chiu , “Clinical Metagenomic Next‐Generation Sequencing for Pathogen Detection,” Annual Review of Pathology 14 (2019): 319–338, 10.1146/annurev-pathmechdis-012418-012751. PMC6345613 30355154

[17] Y. Chen , W. Feng , K. Ye , et al., “Application of Metagenomic Next‐Generation Sequencing in the Diagnosis of Pulmonary Infectious Pathogens From Bronchoalveolar Lavage Samples,” Frontiers in Cellular and Infection Microbiology 11 (2021): 541092, 10.3389/fcimb.2021.541092. 33777827 PMC7991794

[18] T. F. Patterson , G. R. Thompson, 3rd , D. W. Denning , et al., “Practice Guidelines for the Diagnosis and Management of Aspergillosis: 2016 Update by the Infectious Diseases Society of America,” Clinical Infectious Diseases 63 (2016): e1–e60, 10.1093/cid/ciw326. 27365388 PMC4967602

[19] E. Azoulay , L. Russell , A. Van de Louw , et al., “Diagnosis of Severe Respiratory Infections in Immunocompromised Patients,” Intensive Care Medicine 46 (2020): 298–314, 10.1007/s00134-019-05906-5. 32034433 PMC7080052

[20] C. Y. Chiu and S. A. Miller , “Clinical Metagenomics,” Nature Reviews Genetics 20 (2019): 341–355, 10.1038/s41576-019-0113-7. PMC6858796 30918369

[21] X. C. Ji , L. F. Zhou , C. Y. Li , et al., “Reduction of Human DNA Contamination in Clinical Cerebrospinal Fluid Specimens Improves the Sensitivity of Metagenomic Next‐Generation Sequencing,” Journal of Molecular Neuroscience 70 (2020): 659–666, 10.1007/s12031-019-01472-z. 32002752

[22] Q. Miao , Y. Ma , Q. Wang , et al., “Microbiological Diagnostic Performance of Metagenomic Next‐Generation Sequencing When Applied to Clinical Practice,” Clinical Infectious Diseases 67 (2018): S231–S240, 10.1093/cid/ciy693. 30423048

[23] Y. Dong , Q. Chen , B. Tian , J. Li , J. Li , and Z. Hu , “Advancing Microbe Detection for Lower Respiratory Tract Infection Diagnosis and Management With Metagenomic Next‐Generation Sequencing,” Infection and Drug Resistance 16 (2023): 677–694, 10.2147/idr.S387134. 36743335 PMC9896973

[24] B. Goldberg , H. Sichtig , C. Geyer , N. Ledeboer , and G. M. Weinstock , “Making the Leap From Research Laboratory to Clinic: Challenges and Opportunities for Next‐Generation Sequencing in Infectious Disease Diagnostics,” MBio 6 (2015): e01888‐15, 10.1128/mBio.01888-15. 26646014 PMC4669390

[25] C. Langelier , M. S. Zinter , K. Kalantar , et al., “Metagenomic Sequencing Detects Respiratory Pathogens in Hematopoietic Cellular Transplant Patients,” American Journal of Respiratory and Critical Care Medicine 197 (2018): 524–528, 10.1164/rccm.201706-1097LE. 28686513 PMC5821905

[26] X. Wu , Y. Li , M. Zhang , et al., “Etiology of Severe Community‐Acquired Pneumonia in Adults Based on Metagenomic Next‐Generation Sequencing: A Prospective Multicenter Study,” Infectious Diseases and Therapy 9 (2020): 1003–1015, 10.1007/s40121-020-00353-y. 33170499 PMC7652912

[27] Y. Xie , B. Dai , X. Zhou , et al., “Diagnostic Value of Metagenomic Next‐Generation Sequencing for Multi‐Pathogenic Pneumonia in HIV‐Infected Patients,” Infection and Drug Resistance 16 (2023): 607–618, 10.2147/idr.S394265. 36733920 PMC9888013

[28] J. Wang , Y. Han , and J. Feng , “Metagenomic Next‐Generation Sequencing for Mixed Pulmonary Infection Diagnosis,” BMC Pulmonary Medicine 19 (2019): 252, 10.1186/s12890-019-1022-4. 31856779 PMC6921575

[29] H. Xu , X. Hu , W. Wang , et al., “Clinical Application and Evaluation of Metagenomic Next‐Generation Sequencing in Pulmonary Infection With Pleural Effusion,” Infection and Drug Resistance 15 (2022): 2813–2824, 10.2147/idr.S365757. 35677528 PMC9167844

[30] D. Neofytos , C. Hirzel , E. Boely , et al., “ *Pneumocystis jirovecii* Pneumonia in Solid Organ Transplant Recipients: A Descriptive Analysis for the Swiss Transplant Cohort,” Transplant Infectious Disease 20 (2018): e12984, 10.1111/tid.12984. 30155950

[31] G. W. Procop , S. Haddad , J. Quinn , et al., “Detection of *Pneumocystis jiroveci* in Respiratory Specimens by Four Staining Methods,” Journal of Clinical Microbiology 42 (2004): 3333–3335, 10.1128/jcm.42.7.3333-3335.2004. 15243109 PMC446244

[32] J. Jiang , L. Bai , W. Yang , et al., “Metagenomic Next‐Generation Sequencing for the Diagnosis of *Pneumocystis jirovecii* Pneumonia in Non‐HIV‐Infected Patients: A Retrospective Study,” Infectious Diseases and Therapy 10 (2021): 1733–1745, 10.1007/s40121-021-00482-y. 34244957 PMC8322252

[33] D. Wang , S. Fang , X. Hu , et al., “Metagenomic Next‐Generation Sequencing Is Highly Efficient in Diagnosing *Pneumocystis jirovecii* Pneumonia in the Immunocompromised Patients,” Frontiers in Microbiology 13 (2022): 913405, 10.3389/fmicb.2022.913405. 35783441 PMC9247511

[34] J. J. Zhou , W. C. Ding , Y. C. Liu , et al., “Diagnostic Value of Metagenomic Next‐Generation Sequencing for Pulmonary Infection in Intensive Care Unit and Non‐Intensive Care Unit Patients,” Frontiers in Cellular and Infection Microbiology 12 (2022): 929856, 10.3389/fcimb.2022.929856. 36046746 PMC9423675

